# Soluble CD73 in Critically Ill Septic Patients – Data from the Prospective FINNAKI Study

**DOI:** 10.1371/journal.pone.0164420

**Published:** 2016-10-12

**Authors:** Suvi T. Vaara, Maija Hollmén, Anna-Maija Korhonen, Mikael Maksimow, Tero Ala-Kokko, Marko Salmi, Sirpa Jalkanen, Ville Pettilä, FINNAKI Study Group

**Affiliations:** 1 Division of Intensive Care Medicine, Department of Anaesthesiology, Intensive Care and Pain Medicine, University of Helsinki and Helsinki University Hospital, Helsinki, Finland; 2 Medicity Research Laboratory and Department of Medical Microbiology and Immunology, University of Turku, Turku, Finland; 3 Department of Anaesthesiology, University of Oulu and Division of Intensive Care Medicine, Oulu University Hospital, Medical Research Centre Oulu, Oulu, Finland; 4 Department of Intensive Care Medicine, Bern University Hospital (Inselspital), University of Bern, Bern, Switzerland; Kermanshah University of Medical Sciences, ISLAMIC REPUBLIC OF IRAN

## Abstract

**Background:**

CD73 dephosphorylates adenosine monophosphate to adenosine that is an anti-inflammatory molecule inhibiting immune activation and vascular leakage. Therefore, CD73 could be an interesting mediator both in sepsis and acute kidney injury (AKI). We aimed to explore the soluble CD73 (sCD73) levels and their evolution in critically ill patients with severe sepsis and, second, to scrutinize the potential association of sCD73 levels with AKI and 90-day mortality.

**Methods:**

This was a *post-hoc* laboratory analysis of the prospective, observational FINNAKI study conducted in 17 Finnish ICU during 5 months in 2011–2012. Plasma samples of 588 patients admitted with severe sepsis/shock or with developing severe sepsis were analyzed at 0h (ICU admission) and 24h, and additionally, on day 3 or day 5 from a subset of the patients.

**Results:**

The median [IQR] sCD73 levels at 0h were 5.11 [3.29–8.28] ng/mL and they decreased significantly from 0h to 4.14 [2.88–7.11] ng/mL at 24h, *P*<0.001. From 24h to Day 3 (n = 132) the sCD73 levels rose to 5.18 [2.98–8.83] ng/mL (*P* = 0.373) and from 24h to Day 5 (n = 224) to 5.52 [3.57–8.90] ng/mL (*P*<0.001). Patients with AKI had higher sCD73 values at 0h and at 24h compared to those without AKI. Non-survivors with severe sepsis, but not with septic shock, had higher CD73 levels at each time-point compared to survivors. After multivariable adjustments, sCD73 levels at 0h associated independently neither with the development of AKI nor 90-day mortality.

**Conclusions:**

Compared to normal population, the sCD73 levels were generally low at 0h, showed a decrease to 24h, and later an increase by day 5. The sCD73 levels do not seem useful in predicting the development of AKI or 90-day mortality among patients with severe sepsis or shock.

## Background

Severe sepsis is diagnosed up to a third of patients treated in intensive care units (ICUs) [1, 2]. Multiple organ dysfunction (MODS) frequently complicates the course of illness in septic patients and acute kidney injury (AKI) is especially frequent among them [2]. Although the mortality in severe sepsis has showed a decreasing trend [3], it still remains high being 35% within 90-days from ICU admission [2].

The complex pathophysiology of severe sepsis involves both pro-inflammatory reactions to eliminate the invading pathogens and anti-inflammatory responses to limit local and systemic tissue injury [1]. CD73 is an enzyme that dephosphorylates adenosine monophosphate to adenosine, which is a potent anti-inflammatory molecule inhibiting immune activation and vascular leakage [4]. Inhibition of degradation of adenosine decreased leukocyte rolling and adhesion and microvascular dysfunction, and associated with improved survival of septic mice [5]. In further animal models, these effects of adenosine have been shown to occur via CD73 dependent pathways suggesting that CD73-derived adenosine might be beneficial in sepsis [4]. Additionally, CD73 has been suggested to be protective in hypoxia [6] that frequently occurs in the setting of septic shock and MODS. Inflammation can also trigger dysfunction of vascular endothelium increasing tissue edema [7] and CD73 has a key role in re-sealing the endothelial barrier after leukocyte transmigration [6].

CD73 is especially abundant on the cell surfaces in the kidneys [8]. CD73 deficient mice present with proteinuria and deteriorated renal function even in the absence of acute stress factors [9]. Besides alleviating kidney injury in septic mice [4], CD73 has been identified as an endogenous protector from ischemia in the kidneys [8]. In the setting of experimental ischemia-reperfusion injury, the volatile anesthetic isoflurane has been shown to transiently increase the release of active CD73 from endothelial microparticles and to protect from AKI by attenuating the endothelial inflammation and apoptosis [10, 11].

In humans, patients with more severe acute pancreatitis had decreased levels of plasma soluble CD73 (sCD73), and the activity of soluble form of CD73 showed prognostic value in predicting the development of severe acute pancreatitis [12]. Furthermore, interferon-beta treatment that up-regulates CD73, was associated with a reduction in mortality among patients with acute respiratory distress syndrome (ARDS), possibly by decreasing the pulmonary capillary permeability [13]. Capillary leak is a major problem among patients with sepsis [7], and thus, alleviating this phenomenon would be likely to improve the treatment of septic patients.

Therefore, CD73 is an interesting mediator both in sepsis and AKI and a potential target of therapeutic interventions. However, the sCD73 levels among critically ill patients with severe sepsis or AKI are currently unknown. We hypothesized that in severe sepsis and septic shock, the sCD73 concentrations would be decreased–one potential underlying mechanism being increased degradation. Thus, we decided to explore the sCD73 levels and their evolution in critically ill patients with severe sepsis. Moreover, we aimed to scrutinize the potential association of sCD73 levels with AKI and 90-day mortality.

## Methods

This was a *post-hoc* laboratory study of the prospective, observational Finnish Acute Kidney Injury (FINNAKI) study [14] conducted in 17 Finnish intensive care units (ICUs) between September 1, 2011 and February 1, 2012. The Ethics Committee of the Department of Surgery at the Helsinki University Hospital gave nationwide approval for the study protocol that included the analysis of inflammatory and anti-inflammatory biomarkers and thus covered the current analysis. The Ethics Committee approved the use of deferred consent with written, informed consent obtained as soon as possible. Patients enrolled in the study were critically ill, and thus, mostly unable to consent themselves at the time of ICU admission, and contacting patient’s next of kin without delays is not always possible. Consequently, the deferred consent strategy was used to avoid delays in enrolling patients and to allow collecting samples at ICU admission. If the patient was not able to consent due to his/her critical illness, patient’s next of kin was approached as soon as possible to obtain written, informed consent. Laboratory samples were stored and analyzed only if a written, informed consent was obtained from the patient or his/her next of kin. The Finnish National Institute of Health approved data collection from the medical records of deceased patients if informed consent could not be obtained to avoid bias in the primary endpoint of the FINNAKI study, namely the incidence and outcome of AKI [14]. The current laboratory sub-analysis includes only patients who gave or whose next of kin gave a written, informed consent. The study was conducted according to the Declaration of Helsinki.

**Fig 1:**
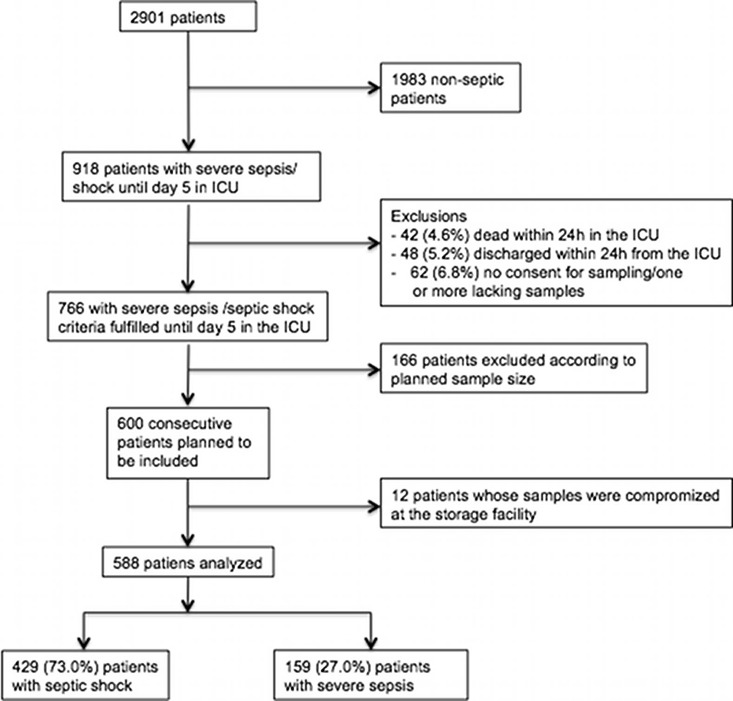
Study flow chart ICU; intensive care unit.

We enrolled all patients with an emergency admission of any duration or an elective post-surgical admission expected to last over 24h in the study. Patients were excluded if they 1) had end-stage renal disease requiring maintenance dialysis, 2) were organ donors, 3) received intermediate care, 4) had received renal replacement therapy (RRT) while enrolled in the study during a previous ICU admission, 5) were transferred from another ICU where the data collection for the study was fulfilled, or 6) were not permanently living in Finland or were unable to give consent due to insufficient language skills. In the current analysis we focused on septic patients and, thus, included patients with blood samples at the ICU admission and at 24h and who fulfilled criteria for severe sepsis/shock [15] within 5 days in ICU [2] (Fig 1).

### Data collection

We recorded patient characteristics, physiological and laboratory data, severity scores, given ICU treatment, diagnoses, and pre-existing comorbidities using case report forms and the ICU data management system via the Finnish Intensive Care Consortium database. Pre-existing chronic comorbidities were deemed present, if mentioned in the patient records. Data were recorded until day 5 if still in ICU. Statistics Finland provided data on survival status at 90 days.

### Definitions

Severe sepsis was defined according to the American College of Chest Physicians/Society of Critical Care Medicine (ACCP/SCCM) definition [15]. Attending clinicians screened the patients for the presence of severe sepsis at the ICU admission and thereafter daily until the diagnostic criteria were fulfilled. We defined the onset of septic shock according to the time of vasopressor initiation (considering norepinephrine, dopamine, or epinephrine). We used Kidney Disease; Improving Global Outcomes (KDIGO) criteria [16] to screen and stage acute kidney injury (AKI) considering both creatinine (measured daily) and urine output (recorded hourly) criteria. Pre-existing chronic comorbidities were defined positive, if mentioned in the patient records. In terms to study the ability of the biomarker to predict AKI, we excluded patients admitted with AKI or AKI diagnosed within the first 12h in the ICU for this particular analysis [2].

### Samples

No previous data on sCD73 levels in septic patients existed to allow a formal sample size calculation. Based on previous studies on novel biomarkers in critically ill septic patients [17, 18], and the known incidence of AKI and 90-day mortality in the FINNAKI population, we decided to study a convenience sample of 600 patients to include a sufficient amount of patients with the main endpoints, namely development of AKI and 90-day mortality [2]. Samples from 12 patients could not be located, and thus, we analyzed plasma samples drawn immediately after ICU admission (0h) and at 24h from 588 consecutive patients with both samples available. Additionally, for patients enrolled before Dec 1, 2011, the study protocol included blood samples drawn on day 3 and on day 5 if still in ICU. Thus, we analyzed samples from patients who had samples available from day 3 or 5 preferring the sample taken on day 5 if both were available. Plasma samples were collected in EDTA tubes, centrifuged, and transferred into plastic tubes and frozen. Samples were kept in -80 degrees until analyzed in August 2015.

### CD73 assays

For the analysis of sCD73 in plasma a sandwich ELISA was performed as previously described [12] with a few modifications. The validation of the new method (DELFIA) is described in detail in the S1 Text Material, Supplementary Methods. To verify that sCD73 levels obtained by DELFIA were indicative of CD73 activity 42 randomly selected patient samples were measured also for CD73 enzyme activity as previously described [19].

### Statistical analyses

We report the non-normally distributed continuous data as median with interquartile range (IQR) and compared it using Mann-Whitney U-test or Kruskal-Wallis test (for comparisons of groups). We report categorical data with count and percentage, and compared it using Fisher’s exact test or Chi-square test where appropriate. Data from two different time-points was compared using Wilcoxon’s signed rank test. We studied correlations of two continuous variables with Spearman’s rho. We assessed the predictive ability of the biomarker by calculating the area under the receiver-operator characteristic curve (AUC). Additionally, we performed univariate modeling for the development of AKI and 90-day mortality, and entered factors with a P-value less than 0.20 in univariate models in multivariable logistic regression to study the independent association of sCD73 levels with these two outcomes. We considered a two-sided p-value less than 0.05 as significant and did not correct for multiple comparisons. All analyses were conducted in SPSS Statistics 20.0 for Mac (IBM, Armonk, NY).

## Results

### Validation of the new CD73 assay and correlation of CD73 activity with sCD73

We found the DELFIA assay for the detection of sCD73 to be reliable, but the absolute values obtained by the DELFIA method were lower than that detected by the ELISA method [12] and therefore cannot be directly compared. However, in a population cohort analyzed with the DELFIA the median [IQR] values for men (n = 1194) were 6.28 ng/ml [5.25–7.66] and for women (n = 1243) 6.46 ng/ml [5.4–8.01]. Also, the sCD73 protein concentration obtained with DELFIA correlated with the CD73 activity (Spearman’s rho 0.838, *P*<0.001) in randomly selected 42 patient samples. The median [IQR] CD73 activity among these 42 patients was 730 [342–1416] nmol/mL/h.

### Study population and sCD73 levels

Samples from 588 consecutive patients were analyzed (study flow chart with exclusions presented as Fig 1). Patients included in the analysis were otherwise representative of the overall cohort with severe sepsis in the FINNAKI study (comparison of patient characteristics presented in the S1 Table), but patients who were not included had higher disease severity, shorter ICU stay, and higher 90-day mortality.

Of the 588 patients, 190 (32.3%) were admitted with septic shock, 300 (51.0%) were admitted with severe sepsis and 179 (59.7%) of them developed septic shock within a median [IQR] of 2.9 [1.7–9.2] hours. Of the 98 (16.7%) patients admitted without fulfilling the criteria for severe sepsis/septic shock, 60 (61.2%) were diagnosed with septic shock after a median [IQR] of 5.4 [2.4–23.7] hours.

Among all patients, the median [IQR] sCD73 levels at 0h were 5.11 [3.29–8.28] ng/mL. Table 1 summarizes the patient characteristics grouped in tertiles according to the sCD73 levels at ICU admission. Patients with pre-existing chronic liver failure (n = 31) had higher sCD73 levels at 0h compared to those without (n = 551), median [IQR] 7.54 [4.08–13.48] vs. 5.04 [3.26–8.20] ng/mL, *P* = 0.014. In comparisons according to the presence of other pre-existing comorbidities (hypertension, chronic obstructive pulmonary disease, arteriosclerosis, diabetes, systolic heart failure, chronic kidney disease, or rheumatoid disease), no differences in sCD73 levels at 0h existed (data not shown).

**Table 1:** Patient characteristics in tertiles according to the sCD73 level at the ICU admission.

	Low	Middle	High	P value^a^
sCD73 –ng/ml	2.83 [2.26–3.30]	5.11 [4.41–6.02]	11.54 [8.25–18.20]	
Age -years	68 [56–77]	63 [48–74]	65 [55–74]	0.121
Male sex	124/196 (63.3%)	126/196 (64.3%)	133/196 (67.9%)	0.395
Hypertension	105/195 (53.8%)	89/195 (45.6%)	102/194 (52.6%)	0.839
Diabetes	46/196 (23.5%)	41/196 (20.9%)	61/196 (31.1%)	0.112
Chronic obstructive pulmonary disease	26/194 (13.4%)	23/193 (11.9%)	21/193 (10.9%)	0.534
Universal arteriosclerosis	29/195 (14.9%)	23/194 (11.9%)	32/193 (16.6%)	0.677
Chronic liver failure	6/195 (3.1%)	8/192 (4.2%)	17/195 (8.7%)	0.030
Chronic kidney disease	13/194 (6.7%)	9/196 (4.6%)	19/195 (9.7%)	0.356
Number of pre-existing comorbidities (0–8)	1 [0–2]	1 [0–2]	1 [0–2]	0.145
Operative admission	72/196 (36.7%)	40/196 (20.4%)	35/196 (17.9%)	<0.001
Mechanical ventilation in ICU	135/196 (68.9%)	138/196 (70.4%)	139/196 (70.9%)	0.741
SAPS II score within 24h (0–163)	40 [32–51]	42 [34–52]	41 [34–56]	0.032
SAPS II score without age points within 24h (0–145)	27 [32–51]	42 [34–52]	41 [34–56]	0.002
SOFA score, first 24h (0–24)	7 [6–10]	8 [6–10]	9 [7–11]	<0.001
Lactate, first in ICU^b^ (mmol/l)	1.5 [1.0–2.5]	1.7 [1.0–2.6]	1.8 [1.1–3.6]	0.027
Fluid balance, cumulative ad day2^c^ -mL	2225 [–29–4292]	1458 [–402–3984]	2230 [–197–4992]	0.477
Septic shock	152/196 (77.6%)	139/196 (70.9%)	138/196 (70.4%)	0.134
Acute kidney injury	90/196 (45.9%)	105/196 (53.6%)	120/196 (61.2%)	<0.003
-stage 1	43 (21.95)	38 (19.4%)	46 (23.5%)	
-stage 2	18 (9.2%)	26 (13.3%)	22 (11.2%)	
-stage 3	29 (14.8%)	41 (20.9%)	52 (26.5%)	
Renal replacement therapy	18/196 (9.2%)	29/196 (14.8%)	41/196 (20.9%)	0.002
Length of ICU stay (days)	4.5 [2.6–8.2]	4.6 [2.7–7.1]	5.0 [3.0–8.7]	0.199
Dead by day 90	45/196 (23.0%)	54/196 (27.6%)	65/196 (33.2%)	0.032

Data presented as median [IQR] or with count/total number and percentage.

^a^P-values are from comparison between the lowest and highest tertile.

^b^ Data missing for 24, 21, and 17 patients

^c^ Data missing for 19, 19, and 13 patients

SAPS; Simplified Acute Physiology Score, SOFA; Sequential Organ Failure Assessment

The sCD73 levels decreased significantly from 5.11 [3.29–8.28] ng/mL at 0h to 4.14 [2.88–7.11] ng/mL at 24h, *P* <0.001. From measurement at 24h to Day 3 (n = 132) the sCD73 levels rose to 5.18 [2.98–8.83] ng/mL (p = 0.373) and from 24h to Day 5 (n = 224) 5.52 [3.57–8.90] ng/mL (*P* <0.001). When patients were grouped in tertiles according to the change in sCD73 values from 0 to 24h, patients in the the lowest tertile (median [IQR] change -3.02 [-5.73–-2.28] ng/ml) had higher lactate and SOFA scores on day 1 compared to those in the highest tertile (median [IQR] change 0.26 [-0.05–1.17] ng/ml) (S2 Table).

### Severity of sepsis and sCD73

**Fig 2:**
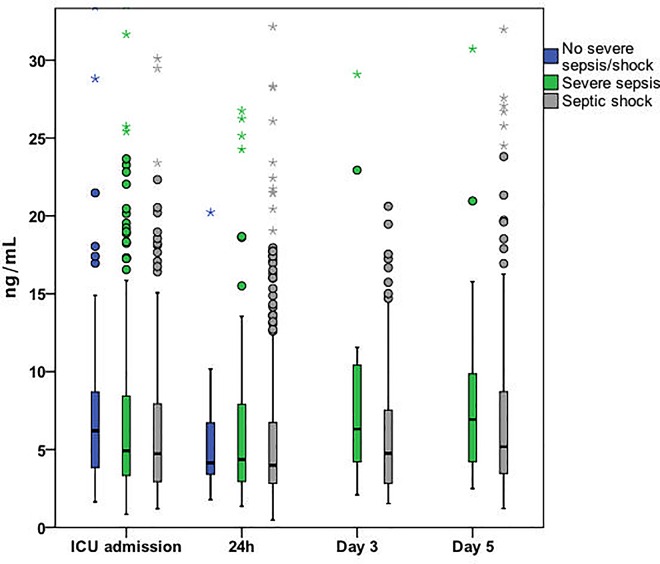
Box-plots presenting sCD73 levels according to the severity of sepsis at the time of sampling. The figure is truncated at level 30 ng/mL level excluding 51 (3.3%) of the cases (range in values 0.47 to 220.59 ng/mL). ICU; intensive care unit. Comparisons: ICU admission: severe sepsis (n = 300) vs. septic shock (n = 190) *P* = 0.158; no sepsis (n = 98) vs. severe sepsis, P = 0.08; no sepsis vs. septic shock *P* = 0.011, across all three groups *P* = 0.031. 24H: severe sepsis (n = 172) vs. septic shock (n = 404) *P* = 0.039; no sepsis (n = 12) vs. severe sepsis, p = 0.980; no sepsis vs. septic shock P = 0.593; across all three groups *P* = 0.196. Day 3: severe sepsis (n = 34) vs. septic shock (n = 98) *P* = 0.014. Day 5: severe sepsis (n = 44) vs. septic shock (n = 180) *P* = 0.086.

Fig 2 presents sCD73 levels in all time points according to the severity of sepsis. Patients admitted without severe sepsis/shock had significantly higher sCD73 levels compared to those admitted with severe sepsis or shock. Patients with severe sepsis did not differ from those with septic shock at 0h, but at 24h and on day 3 their sCD73 levels were higher. sCD73 level at 0h did not predict the development of septic shock among those 398 patients admitted without shock, the AUC was 0.47 (95% CI 0.42–0.53). The sCD73 levels at different time-points did not correlate with the severity of sepsis at that time-point (intraclass correlation -0.492; 95% CI -0.493–-0.491, *P*>0.999).

### Acute kidney injury and sCD73

Patients with AKI diagnosed until day 5 in the ICU had higher sCD73 values at 0h and at 24h compared to those without AKI (Table 2). When patients admitted with AKI (AKI diagnosis <12h from ICU admission, n = 179) were excluded, the sCD73 levels at 0h but not at 24h were significantly higher. After adjusting for confounders, sCD73 level at 0h categorized in tertiles was not significantly associated with an increased risk of development of AKI Table 3. Patients who eventually received RRT had higher CD73 levels at 0h (when not yet in RRT), *P* = 0.004, but not in other time-points (*P* = 0.072 at 24h, 0.171 on day 3, and 0.343 on day 5).

**Table 2:** Soluble CD73 concentrations (ng/mL) according to the presence/development of acute kidney injury (AKI).

	All AKI (n = 315)	No AKI (n = 273)	P^a^	AKI >12h from ICU admission (n = 136)	*P*-value^b^
-0h	5.67 [3.44–9.30]	4.55 [3.20–7.29]	0.004	5.48 [3.56–8.51]	0.040
-24h	4.50 [2.95–7.67]	3.91 [2.80–6.62]	0.044	4.28 [3.06–7.44]	0.157
-Decrease from 0h to 24h	0.86 [0.11–2.27]	0.67 [0.04–1.69]	0.106	0.79 [0.15–2.29]	0.121
Day 3^c^	5.47 [2.95–9.52]	4.94 [2.99–7.25]	0.484	4.12 [2.42–7.68]	0.580
Day 5^d^	5.71 [3.57–9.07]	5.23 [3.57–8.32]	0.618	5.64 [3.76–9.14]	0.484

^a^ Comparison between all AKI and non-AKI patients

^b^ Comparison between AKI>12h and non-AKI patients

^c^ Number of patients: All AKI 75, No AKI 57, AKI >12h 25

^d^ Number of patients: All AKI 123, No AKI 101, AKI >12h 61

**Table 3:** Univariate and multivariable logistic regression models for the development of AKI.

	Univariate OR (95% CI)	P-value	Multivariate OR (95% CI)	P-value
sCD73 0h –ng/ml	1.01 (0.99–1.03)	0.266		
sCD73 0h –categorized in tertiles	1.30 (1.00–1.68)	0.046	1.32 (0.98–1.78)	0.070
Age	1.02 (1.01–1.03)	0.004	1.02 (1.00–1.03)	0.025
Male gender	0.89 (0.58–1.37)	0.590		
Hypertension	1.36 (0.90–2.06)	0.141	1.05 (0.63–1.74)	0.868
Diabetes	1.56 (0.97–2.50)	0.067	1.19 (0.67–2.10)	0.560
Chronic obstructive pulmonary disease	0.52 (0.26–1.06)	0.070	0.43 (0.20–0.93)	0.032
Universal arteriosclerosis	0.92 (0.50–1.70)	0.788		
Chronic liver failure	1.00 (0.40–2.55)	0.993		
Chronic kidney disease	4.42 (1.84–10.60)	0.001	4.11 (1.50–11.23)	0.006
Operative admission	1.41 (0.89–2.24)	0.145	1.01 (0.58–1.77)	0.970
Vasoactive drugs on day 1	2.38 (1.49–3.82)	<0.001	0.77 (0.36–1.62)	0.485
Mechanical ventilation in ICU	1.85 (1.14–2.99)	0.013	1.56 (0.85–2.84)	0.149
SAPS II score without age and renal points within 24h	1.29 (1.14–1.46)	<0.001	0.99 (0.97–1.01)	0.382
Lactate, first in ICU^b^ (mmol/l)^a^	1.29 (1.24–1.46)	<0.001	1.21 (1.07–1.37)	0.002
Fluid balance, cumulative ad day2^c^ –mL–categorized quintiles^b^	1.47 (1.20–1.81)	<0.001	1.24 (0.99–1.55)	0.066
Septic shock	2.82 (1.71–4.64)	<0.001	3.02 (1.38–6.59)	0.006

ICU; Intensive care unit, CI; confidence interval, OR; odds ratio, SAPS; Simplified Acute Physiology Score. Number of patients included in the multivariable model was 402 (0f 409). Hosmer-Lemeshow Chi-Square 9.12, P = 0.332.

^a^42 missing values imputed with the median value for the multivariable model

^b^38 missing values imputed with the median value for the multivariable model

### 90-day mortality and sCD73

Of the 588 patients, 164 (27.9%; 95% CI from 24.3% to 31.5%) were dead by day 90. The non-survivors had significantly higher sCD73 levels both at 0h and at 24h (Table 4). The difference remained significant, when patients with AKI were excluded. When patients with severe sepsis and septic shock were analyzed separately, the difference between survivors and non-survivors was seen only among those with severe sepsis. After adjusting for several confounders, sCD73 level at 0h was not associated with an increased risk for 90-day mortality (Table 5). Patients did not differ in terms of 90-day mortality when compared in groups according to the change in sCD73 levels between 0 and 24h (Additional File Table 2).

**Table 4:** Soluble CD73 concentrations (ng/mL) according to the 90-day survival.

	N	90-day survivors	N	90-day non-survivors	*P*-value
All patients					
-0h	424	4.87 [3.21–7.93]	164	5.73 [3.65–10.25]	0.018
-24h	424	3.96 [2.83–6.71]	164	4.80 [3.07–8.73]	0.013
-Day 3	97	4.98 [2.77–7.86]	35	6.05 [3.95–10.43]	0.115
-Day 5	151	5.30 [3.49–8.88]	73	5.80 [3.67–8.98]	0.995
Severe sepsis					
-0h	121	4.91 [3.31–7.31]	38	8.15 [4.59–15.00]	0.001
-24h	121	4.09 [2.88–7.02]	38	6.69 [3.77–11.71]	0.005
-Day 3	25	5.74 [3.04–9.75]	8	9.34 [6.41–32.94]	0.026
-Day 5	30	7.05 [4.09–9.64]	14	6.41 [4.02–11.00]	>0.999
Septic shock					
-0h	303	4.83 [3.12–8.12]	126	5.15 [3.30–8.83]	0.330
-24h	303	3.90 [2.80–6.68]	126	4.37 [2.87–8.05]	0.168
-Day 3	72	4.82 [2.62–7.23]	27	4.62 [3.20–9.17]	0.535
-Day 5	121	5.14 [3.43–8.98]	59	5.57 [3.66–8.44]	0.971
Patients without acute kidney injury					
-0h	215	4.31 [3.01–6.89]	58	5.30 [3.60–11.13]	0.009
-24h	215	3.77 [2.76–6.30]	58	4.43 [3.12–9.62]	0.018
-Day 3	49	4.84 [2.85–6.61]	8	7.68 [4.29–24.43]	0.073
-Day 5	69	4.61 [3.24–7.97]	32	6.37 [4.21–10.62]	0.049

Data presented as median [IQR]

**Table 5:** Univariate and multivariable logistic regression models for 90-day mortality.

	Univariate OR (95% CI)	P-value	Multivariate OR (95% CI)	P-value
sCD73 0h –ng/ml	1.01 (0.99–1.02)	0.331		
sCD73 0h –categorized in tertiles	1.29 (1.03–1.61)	0.025	1.19 (0.92–1.53)	0.177
Age	1.04 (1.03–1.05)	<0.001	1.05 (1.03–1.07)	<0.001
Male gender	1.26 (0.86–1.86)	0.234		
Hypertension	1.36 (0.94–1.95)	0.101	0.90 (0.58–1.38)	0.623
Diabetes	0.86 (0.56–1.31)	0.487		
Chronic obstructive pulmonary disease	1.13 (0.66–1.96)	0.655		
Universal arteriosclerosis	1.36 (0.83–1.23)	0.225		
Chronic liver failure	2.96 (1.43–6.14)	0.004	4.31 (1.86–9.99)	0.001
Chronic kidney disease	1.95 (1.02–3.73)	0.044	1.67 (0.82–3.42)	0.160
Operative admission	0.59 (0.38–0.92)	0.020	0.49 (0.28–0.83)	0.008
Vasoactive drugs on day 1	1.28 (0.85–1.93)	0.234		
Mechanical ventilation in ICU	1.84 (1.20–2.81)	0.005	1.43 (0.84–2.41)	0.186
SAPS II score without age and renal points within 24h	1.04 (1.03–1.06)	<0.001	1.04 (1.02–1.06)	<0.001
Lactate, first in ICU^b^ (mmol/l)^a^	1.05 (0.99–1.12)	0.108	0.97 (0.90–1.05)	0.513
Fluid balance, cumulative ad day2^c^ –mL–categorized in kvintiles^b^	1.28 (1.08–1.52)	0.005	1.20 (0.99–1.46)	0.060
Septic shock	1.32 (0.87–2.01)	0.190	1.00 (0.61–1.64)	0.993

ICU; Intensive care unit, CI; confidence interval, OR; odds ratio, SAPS; Simplified Acute Physiology Score. Number of patients included in the multivariable model was 573. Hosmer-Lemeshow Chi-Square 10.93, P = 0.206.

^a^ 62 missing values imputed with the median value for the multivariable model

^b^ 51 missing values imputed with the median value for the multivariable model

## Discussion

In this sub-cohort of critically ill septic patients from the prospective FINNAKI study we found that the sCD73 levels were generally low at 0h compared to previously reported data, showed a decrease to 24h, and an increase by day 5. Patients admitted with septic shock presented with the lowest concentrations. Moreover, patients with AKI and 90-day non-survivors with severe sepsis presented with slightly higher levels compared to non-AKI patients and 90-day non-survivors, but the sCD73 levels were not independently associated with the development of AKI or 90-day mortality.

The origin of sCD73 measured in plasma is thought to be mainly from shedding from blood lymphocytes [20]. Compared to the values obtained in a population cohort using the same analysis method, and previously measured in healthy volunteers and patients with acute pancreatitis with another method [12], the sCD73 levels in this cohort of critically ill patients were low. A population cohort study has reported patients with peripheral arterial disease involving chronic inflammation and hypoxia to present with increased CD73 activity [21]. We did not find the sCD73 levels in patients with COPD or arteriosclerosis to be increased, but found patients with chronic liver failure to have increased levels, likely because sCD73 levels have been found to be confounded by elevated plasma alkaline phosphatase levels [21]. Regarding the severity of critical illness, our findings corroborate results from patients with acute pancreatitis [12] in that patients with the most severe form of sepsis had the lowest sCD73 levels.

Majority of patients showed a decrease in sCD73 levels within the 24h in ICU. By day 5, we demonstrated an increase in sCD73 levels. The drop seen at 24h could be due to increased degradation of sCD73 or its leakage to the tissues. Unfortunately the lymphocyte levels were not measured so that we cannot comment on whether the initial drop seen in sCD73 levels would have been due to a reduction in circulating lymphocyte levels and decreased shedding of sCD73. Patients categorized according to the magnitude of change in sCD73 levels between 0 and 24h did not differ regarding fluid balance, and, thus, the observed drop is unlikely to be due to hemodilution. Alternatively, CD73 production may be diminished like reported to occur in leukocytes of patients with severe pancreatitis and low levels of sCD73 [12]. As CD73 dephosphorylates adenosine monophosphate into adenosine [6], our findings of a decrease in sCD73 levels by 24h are partly in line with a study demonstrating that adenosine levels decreased from the beginning of shock until 72h [22].

Patients with AKI presented with higher sCD73 levels at 0h and 24h. This is surprising, as one could assume AKI to involve increased degradation of sCD73. Unfortunately, no studies examining the sCD73 levels and AKI in the critically ill exist for comparison. Potentially, endogenous mechanisms are working to increase the sCD73 levels given that work in mice has shown induction of CD73 to be protective in ischemia-reperfusion–related AKI [10]. Decreased plasma clearance of sCD73 in severe AKI might also explain the finding. The molecular weight of sCD73 is about 70kDa [20], which exceeds the cut-off of modern filters used in continuous RRT machines, and among patients with RRT sCD73 could accumulate. However, no difference between the patients with and without RRT was observed except at 0h, when RRT was not yet commenced. Finally, when adjusted for a number of confounders, the sCD73 levels at ICU admission were not independently associated with the development of AKI, indicating that sCD73 is probably not useful in predicting the development of AKI.

We found the non-survivors with severe sepsis but not with septic shock to have higher sCD73 levels compared to survivors. Patients with AKI also had higher sCD73 levels, which could potentially also associate with worse outcome. However, the difference between survivors and non-survivors regardless of the severity of sepsis was robust for exclusion of AKI patients, suggesting that decreased clearance due to AKI or AKI *per se* do not explain these findings. If sCD73 levels in plasma are solely from circulating lymphocytes [20], then the increased levels seen among the non-survivors could reflect a more pronounced response to infection and potentially, also more severe infection. This, however, is unlikely, because patients with septic shock had lower sCD73 levels. Overall, the differences between survivors and non-survivors were small in magnitude, and were not independently associated with 90-day mortality after adjusting for confounders. Therefore, sCD73 does not seem to be a useful general biomarker for predicting mortality. Possibly, more frequent sampling would help to reveal whether there are detectable short-term trends in the sCD73 levels that would further help to reveal the complex pathophysiology behind these syndromes.

The encouraging results of using interferon-beta to increase the activity of CD73, and potentially, decrease the permeability of pulmonary capillaries, showed that among patients with ARDS the treatment increased the CD73 activity almost to three-fold during the dosing period [13]. In a sub-analysis of the current analysis, we found that sCD73 levels correlated with the activity of CD73 and the median baseline level of CD73 activity was higher compared to the baseline of the ARDS cohort [13]. Albeit the non-survivors in our study with severe sepsis had higher sCD73 levels compared to survivors, the differences were generally very small and not independently associated with an increased risk for mortality. Most notably, no difference in sCD73 among patients with septic shock was seen at any time-point. Therefore, our results do not exclude the possibility that induction of CD73 could turn out to be useful also among patients with septic shock in future studies as has been suggested for ARDS [13].

The strengths of our analysis include detailed collection of clinical data and the multicenter design. Several limitations should be discussed, however. First, to study the trend in sCD73 levels, we analyzed samples from patients of whom both 0h and 24h plasma were available. Therefore, those who died or were discharged shortly after admission were excluded (9.8% of the severe sepsis FINNAKI cohort) [2]. Inclusion of those with imminent death or very short ICU stay indicating rapid improvement of clinical status would not have served the aims of this study, however, as these subjects are unlikely to benefit from predictive biomarkers. Second, analyzing samples with more frequent sampling might have helped to better detect the kinetics of sCD73 regarding the development of septic shock. Third, we did not analyze samples from non-septic controls, which might have helped us to separate the effects of critical illness as such and infection *per se*. Fourth, the differences between all groups in the sCD73 concentrations were generally small, and the clinical significance of these is unclear.

## Conclusions

In conclusion, in this *post-hoc* laboratory analysis prospective, multicenter FINNAKI study among septic critically ill patients, we found that the sCD73 levels were generally low and showed a further decrease from 0h to 24h. Moreover, the sCD73 levels were higher in AKI versus non-AKI patients and in non-survivors with severe sepsis than in survivors, but were not independently associated either with the development of AKI or 90-day mortality. The sCD73 levels do not seem useful in predicting the outcome of patients with severe sepsis or shock.

## Supporting Information

S1 Dataset CD73 values, patient characteristics and study endpoints. (XLSX)

S1 Table Comparison of patients included in the current laboratory analysis to those not included from the FINNAKI severe sepsis/shock cohort. (PDF)

S2 Table Patient characteristics and outcomes according to the change in sCD73 values between 0h and 24h classified in tertiles. (PDF)

S1 Text Material Supplementary methods. (PDF)
