# Supplementary material for: Soluble CD73 in Critically Ill Septic Patients – Data from the Prospective FINNAKI Study
Source: PLoS One. 2016 Oct 12;11(10):e0164420. doi: 10.1371/journal.pone.0164420 (PMC5061395; doi:10.1371/journal.pone.0164420)
Supplement: S1 Table — (PDF) [file pone.0164420.s002.pdf]

Additional File Table 1. Comparison of patients included in the current laboratory analysis to those not included from the FINNAKI severe sepsis/shock cohort.

	Included N=588	Not included N=330	P-value
Age	65 [53-75]	65 [55-75]	0.762
Male sex	383/588 (65.1%)	206/330 (62.45)	0.430
Hypertension	296/584 (50.7%)	183/327 (56.0%)	0.129
Diabetes	148/588 (25.2%)	81/330 (24.5%)	0.874
Chronic obstructive pulmonary disease	70/588 (12.1%)	41/327 (12.5%)	0.834
Universal arteriosclerosis	84/584 (14.4%)	48/322 (14.9%)	0.845
Chronic liver failure	31/582 (5.3%)	16/325 (4.9%)	0.876
Chronic kidney disease	41/585 (7.0%)	19/328 (5.8%)	0.578
Operative admission	147/588 (25.0%)	76/330 (23.0%)	0.522
Vasoactive drugs on day 1	420/588 (71.4%)	241/330 (73.0%)	0.646
Mechanical ventilation in ICU	412/588 (70.1%)	237/330 (71.8%)	0.597
SAPS II score within 24h (0-163)	41 [33-53]	45 [34-59]	0.001
SAPS II score without age points within 24h (0-145)	30 [23-40]	33 [23-48]	0.002
SOFA score, first 24h (0-24)	8 [6-10]	8 [6-11]	0.024
Lactate, first in ICU[a] (mmol/L)	1.6 [1.1-3.0]	2.1 [1.1-4.4]	<0.001
Acute kidney injury	315/588 (53.6%)	173/330 (52.4%)	0.784
-stage 1	127 (21.6%)	67 (20.3%)	
-stage 2	66 (11.2%)	31 (9.4%)	
-stage 3	122 (20.7%)	75 (22.7%)	
Renal replacement therapy	88/588 (15.0%)	53/330 (16.1%)	0.703
Length of ICU stay (days)	4.7 [2.8-8.1]	2.8 [0.9-5.9]	<0.001
Dead by day 90	164/588 (27.9%)	128/330 (38.8%)	0.001

Data presented as median [IQR] or with count/total number and percentage.

[a] Data missing for 62 and 82 patients

SAPS; Simplified Acute Physiology Score, SOFA; Sequential Organ Failure Assessment
