# Supplementary material for: Soluble CD73 in Critically Ill Septic Patients – Data from the Prospective FINNAKI Study
Source: PLoS One. 2016 Oct 12;11(10):e0164420. doi: 10.1371/journal.pone.0164420 (PMC5061395; doi:10.1371/journal.pone.0164420)
Supplement: S2 Table — (PDF) [file pone.0164420.s003.pdf]

Additional File Table 2. Patient characteristics and outcomes according to the change in sCD73 values between 0h and 24h classified in tertiles.

	Lowest (n=168)	Middle (n=225)	High (n=195)	P-value[a]
CD73 0h -ng/ml	11.13 [7.04-17.94]	4.52 [3.33-6.01]	3.31 [2.43-5.62]	
CD73 24h –ng/ml	7.02 [4.46-11.74]	3.58 [2.66-4.83]	3.70 [2.72-6.75]	
Change from 0h to 24h –ng/ml	-3.02 [-5.73- -2.28]	-0.82 [-1.19- -0.52]	0.26 [-0.05-1.17]	
Age	65 [52-74]	65 [54-76]	65 [54-74]	0.766
Male sex	112/168 (66.7%)	152/225 (67.6%)	119/195 (61.0%)	0.276
Chronic obstructive pulmonary disease	19/165 (11.5%)	26/222 (11.7%)	25/193 (13.0%)	0.748
Hypertension	78/166 (47.0%)	112/225 (49.8%)	106/193 (54.9%)	0.140
Diabetes	43/168 (25.6%)	53/225 (23.6%)	52/195 (26.7%)	0.905
Universal arteriosclerosis	29/165 (17.6%)	31/224 (13.8%)	24/193 (12.4%)	0.182
Chronic liver failure	10/165 (6.1%)	13/222 (5.9%)	8/195 (4.1%)	0.470
Chronic kidney disease	13/168 (7.7%)	16/224 (7.1%)	12/193 (6.2%)	0.679
Number of pre-existing comorbidities	1 [0-2]	1 [0-2]	1 [0-2]	0.697
Operative admission	34/168 (20.2%)	57/225 (25.3%)	56/195 (28.7%)	0.068
Mechanical ventilation in ICU	127/168 (75.6%)	157/225 (69.5%)	128/195 (65.6%)	0.050
Septic shock	126/168 (75.0%)	157/225 (69.8%)	146/195 (74.9%)	>0.999
SAPS II score within 24h (0-163)	43 [34-56]	40 [33-52]	40 [33-51]	0.084
SOFA score, first 24h (0-24)	9 [7-11]	8 [5-10]	8 [6-10]	0.008
Lactate, first in ICU (mmol/L)[b]	1.9 [1.2-3.8]	1.6 [1.1-2.5]	1.5 [1.0-3.0]	0.016
Fluid balance, d1[c]	1270 [22-3018]	985 [0-2838]	676 [-180-2540]	0.068
Fluid balance, cumulative ad day 2 –mL[d]	2557 [-322-5710]	1718 [-103-3820]	1737 [-314-4194]	0.056
Acute kidney injury	101/168 (60.1%)	112/225 (49.8%)	102/195 (52.3%)	0.139
Renal replacement therapy	31/168 (18.5%)	28/225 (12.4%)	29/195 (14.9%)	0.397
Dead by day 90	53/168 (31.5%)	57/225 (24.3%)	54/195 (27.7%)	0.489

Data presented as median [IQR] or with count/total number and percentage.

SAPS; Simplified Acute Physiology Score, SOFA; Sequential Organ Failure Assessment

[a] P-values are from comparison between the lowest and highest tertile.

[b] Values from from 19, 23, and 20 patients were missing

[b] Values from 8, 20, and 18 patients were missing

[d] Values from 9, 24, and 18 patients were missing
