# Supplementary material for: Soluble CD73 in Critically Ill Septic Patients – Data from the Prospective FINNAKI Study
Source: PLoS One. 2016 Oct 12;11(10):e0164420. doi: 10.1371/journal.pone.0164420 (PMC5061395; doi:10.1371/journal.pone.0164420)
Supplement: S1 Text Material — (PDF) [file pone.0164420.s004.pdf]

Supplementary Methods

Validation of the new assay for the detection of soluble CD73

We developed a new modification of our published method[1] to increase the sensitivity of measured sCD73. For the analysis of sCD73 in plasma a sandwich ELISA was performed as previously described[1] with a few modifications. Pooled plasma from six healthy donors (5 female, 1 male) from which CD73 was depleted by three sequential incubations with CnBr-activated sepharose beads armed with an anti-CD73 mAb 4G4 (Invivo Biotech) was used together with recombinant human CD73 (rh5`-nucleotidase, 5795-EN; R&D Systems) to create a standard curve for the DELFIA assay. The recombinant CD73 was dissolved in 20mM NaCl, 4mM $CaCl_2$ and 20%v/v glycerol and diluted in CD73-depleted plasma 1:20 in PBS before use. The CD73 signal was developed using Eu-Labelled Streptavidin (1244-360), Delfia Assay Buffer (1244-111) and Delfia Enhancement Solution (1244-105; all from Perkin Elmer) according to the manufacturer's instructions. The europium was measured with a Multilabel Counter 1420 VICTOR3 (Perkin Elmer) using time-resolved fluorescence.

The detection limit of the new assay (defined as 3xSD) using non-depleted plasma pool as inter and intra assay control was 0.072 [0.065-0.08] ng/mL and the quantification limit (defined as 10xSD) was 0.097 [0.076-0.12] ng/mL (inter CV% 4.763 %; intra CV% 2.71 [1.035-5.165] %). Since the europium-based assay gave generally lower sCD73 concentrations as compared to the levels obtained from the serum of acute pancreatitis patients using a chemiluminescence-based ELISA[1] we tested the sCD73 levels of nine patients from the Maksimow et al. study[1] with the DELFIA method using CD73 standard diluted in CD73-depleted serum. Of note, the matrix of the samples (serum vs. plasma) did not affect the detected sCD73 levels since the standard curves created with both matrices gave equal CD73 values (Pearson r = 1). The mean CD73 level for the analysed acute pancreatitis patients obtained by our assay was 16.1 [11.2-20.9] ng/mL compared to the mean 78.4 [51.7-105.1] ng/mL reported for patients with standard ELISA. Although, the

DELFIA detected a lower CD73 level in the same patient, the two methods correlated in detecting the high and low CD73 patients R^2=0.9, P<0.0001. Importantly, the values for 280 and 458 FINNAKI patients would have not been detected and quantified, respectively, with standard ELISA since the reported detection limit of the ELISA method in the Maksimow et al. study[1] was approximately 5 ng/mL and quantitation limit was 9 ng/mL. Significantly lower detection limits, a broader range and lower absolute values for DELFIA than for ELISA have been reported.[2 3] We, thus, conclude that our assay is reliable but the absolute values obtained by these two methods cannot be directly compared.

References:

1. Maksimow M, Kyhälä L, Nieminen A, Kylänpää L, Aalto K, Elima K, et al. Early prediction of persistent organ failure by soluble CD73 in patients with acute pancreatitis*. *Crit Care Med* 2014; **42**: 2556-64
2. Bonin E, Tiru M, Hallander H, Bredberg-Raden U. Evaluation of single- and dual antigen delayed fluorescence immunoassay in comparison to an ELISA and the in vivo toxin neutralisation test for detection of diphtheria toxin antibodies. *J Immunol Methods* 1999; **230**: 131-40
3. Cowans NJ, Kisanga M, Khan A, Spencer K. A comparison of two immunoassay methods for the measurement of maternal serum placental growth factor in early pregnancy. *Fetal Diagn Ther* 2012; **31**: 254-9
